# Artificial neural networks: powerful tools for modeling chaotic behavior in the nervous system

**DOI:** 10.3389/fncom.2014.00040

**Published:** 2014-04-09

**Authors:** Malihe Molaie, Razieh Falahian, Shahriar Gharibzadeh, Sajad Jafari, Julien C. Sprott

**Affiliations:** ^1^Department of Bioelectric, Biomedical Engineering Faculty, Amirkabir University of TechnologyTehran, Iran; ^2^Department of Physics, University of WisconsinMadison, Wisconsin, WI, USA

**Keywords:** artificial neural networks, biological systems, electroretinogram, chaos, bifurcation diagram

Modeling real-world systems plays a pivotal role in their analysis and contributes to a better understanding of their behavior and performance. Classification, optimization, control, and pattern recognition problems rely heavily on modeling techniques. Such models can be categorized into three classes: white-box, black-box, and gray-box (Nelles, 2001). White-box models are fully derived from first principles, i.e., physical, chemical, biological, economical, etc. laws. All equations and parameters are determined from theory. Black-box models are based solely on experimental data, and their structure and parameters are determined by experimental modeling. Building black-box models requires little or no prior knowledge of the system. Gray-box models represent a compromise or combination of white-box and black-box models (Nelles, 2001).

In the modeling of highly nonlinear and complex phenomena, we may not have a good understanding of the processes, and thus black-box models may be our best (or even our only) choice. Artificial neural networks (ANNs) are one of the most powerful and popular tools for black-box modeling and are designed and inspired by real biological neural networks.

There has been an increasing interest in analyzing neurophysiology from a nonlinear and chaotic systems viewpoint in recent years (Christini and Collins, 1995; Sarbadhikari and Chakrabarty, 2001; Korn and Faure, 2003; Hadaeghi et al., 2013; Jafari et al., 2013; Mattei, 2013). For example, although the famous Hodgkin and Huxley model (Hodgkin and Huxley, 1952) has been the basis of almost all of the proposed models for neural firing, the Rose-Hindmarsh model (Hindmarsh and Rose, 1984) is known to be a more refined model since it can show different firing patterns, especially chaotic bursts of action potential, which enable a proper matching between this model behavior and experimental data. Another example of the observation of chaotic behavior in the nervous system is the period-doubling route to chaos in flicker vision (Crevier and Meister, 1998), which is the focus of this letter.

Stimulation with periodic flashes of light is useful for distinguishing some disorders of the human visual system (Crevier and Meister, 1998). It has been shown by Crevier and Meister (1998) that during electroretinogram (ERG) recordings of the visual system, period-doubling can occur. It is well-known that period-doubling occurs in nonlinear dynamical systems, and it is often associated with the onset of chaos. In one study (Crevier and Meister, 1998) the retina of a salamander was stimulated with a periodic square-wave flashes, and the ERG was recorded. The flash frequency was changed between zero and 30 Hz, while the contrast was constant. In another record, the contrast was changed while the frequency was fixed at 16 Hz. All the ERG signals were filtered at 1–1000 Hz. Using a common approach to obtain a discrete time series from a continuous recorded signal, successive local maxima of the signal were extracted as a time series (Figure 1A). As shown in Figures 1B,C, both the parameters (flash frequency and contrast) have a great effect on the recorded ERG signals and cause bifurcations resulting in a period-doubling route to chaos.

**Figure 1:**
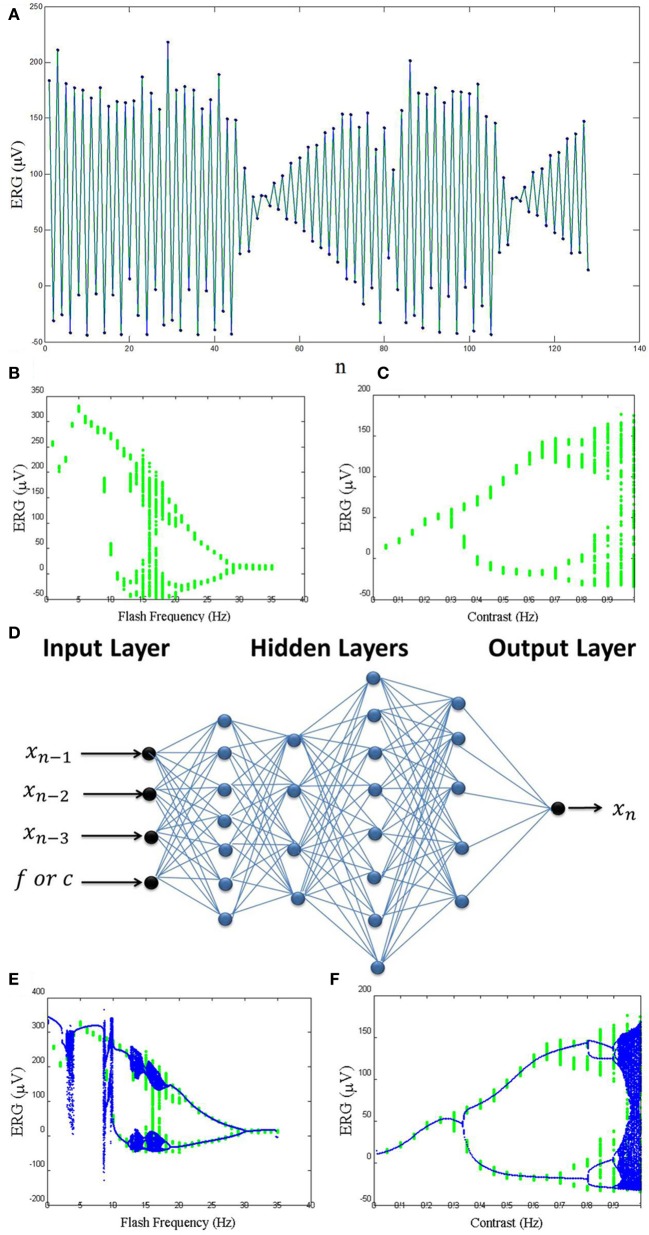
**(A)** One example of the local maxima of the ERG signals. **(B)** Real bifurcation diagram resulted from varying flash frequency. **(C)** Real bifurcation diagram resulted from varying contrast. **(D)** The structure of the ANNs that were used. **(E)** Artificial bifurcation diagram resulted from varying the flash frequency input in the ANN. **(F)** Artificial bifurcation diagram resulted from varying the contrast input in the ANN.

However, it is difficult to understand the exact relations between the parameters and their effects. In other words, it is not easy to build a white-box model that can regenerate the signals and diagrams accurately. That may be because of the highly complex and nonlinear dynamics involved. We have used the ability of an ANN in learning highly nonlinear dynamics as a black-box model of this system. We used a four hidden layer feed-forward neural network with (7/4/8/5) neurons in the layers (Figure 1D) and nonlinear transfer functions hyperbolic tangent function that help the network learn the complex relationships between input and output. The activation function of the last layer of the network is linear transfer function. We used two parameters (contrast and frequency) and three time delays (*x_n_*_−1_, *x_n_*_−2_, and *x_n_*_−3_) as the inputs of the ANN to fit each data point of the time series (*x_n_*) as the output of the network.

As shown in Figures 1E,F, this model can generate bifurcation diagrams similar to those obtained from real data. As the result, we believe that ANNs are powerful tools for modeling highly nonlinear behavior in the nervous system. We plan to construct ANN models in future work including extension to more cases and details, extension of the ideas in Hadaeghi et al. (2013) to patients with bipolar disorder, and extension of the ideas in Jafari et al. (2013) to patients with attention deficit hyperactivity disorder (ADHD).

## Conflict of interest statement

The authors declare that the research was conducted in the absence of any commercial or financial relationships that could be construed as a potential conflict of interest.
